# Fusion of Appearance and Motion Features for Daily Activity Recognition from Egocentric Perspective

**DOI:** 10.3390/s23156804

**Published:** 2023-07-30

**Authors:** Mohd Haris Lye, Nouar AlDahoul, Hezerul Abdul Karim

**Affiliations:** 1 Faculty of Engineering, Multimedia University, Cyberjaya 63100, Selangor, Malaysia; hezerul@mmu.edu.my; 2 Computer Science, New York University, Abu Dhabi P.O. Box 1291888, United Arab Emirates

**Keywords:** activities of daily living, convolutional neural network, egocentric vision, feature fusion, optical flow

## Abstract

Vidos from a first-person or egocentric perspective offer a promising tool for recognizing various activities related to daily living. In the egocentric perspective, the video is obtained from a wearable camera, and this enables the capture of the person’s activities in a consistent viewpoint. Recognition of activity using a wearable sensor is challenging due to various reasons, such as motion blur and large variations. The existing methods are based on extracting handcrafted features from video frames to represent the contents. These features are domain-dependent, where features that are suitable for a specific dataset may not be suitable for others. In this paper, we propose a novel solution to recognize daily living activities from a pre-segmented video clip. The pre-trained convolutional neural network (CNN) model VGG16 is used to extract visual features from sampled video frames and then aggregated by the proposed pooling scheme. The proposed solution combines appearance and motion features extracted from video frames and optical flow images, respectively. The methods of mean and max spatial pooling (MMSP) and max mean temporal pyramid (TPMM) pooling are proposed to compose the final video descriptor. The feature is applied to a linear support vector machine (SVM) to recognize the type of activities observed in the video clip. The evaluation of the proposed solution was performed on three public benchmark datasets. We performed studies to show the advantage of aggregating appearance and motion features for daily activity recognition. The results show that the proposed solution is promising for recognizing activities of daily living. Compared to several methods on three public datasets, the proposed MMSP–TPMM method produces higher classification performance in terms of accuracy (90.38% with LENA dataset, 75.37% with ADL dataset, 96.08% with FPPA dataset) and average per-class precision (AP) (58.42% with ADL dataset and 96.11% with FPPA dataset).

## 1. Introduction

Activities of daily living (ADL) are a type of activity performed daily and repetitively. ADL relates to the personal tasks performed to sustain healthy living. The types of activity include eating, food preparation, reading, and dressing. In the medical literature, ADL is categorized according to its functional purpose. In the work by [1], a hierarchical division of activities is used. At the highest level, ADL is divided into hygiene, food-related, and entertainment. It is of interest to monitor a person’s ADL if they are afflicted by disease or due to aging factors. Such factors cause ADL impairment that warrants close supervision by doctors and their caretakers.

In smart homes, vision-based activity recognition is used to observe the person’s image and predict their actions. This helps in several aspects, such as entertainment (playing favorite music, turning on TV channel, turning on the Aircon), daily assistance (turning on/off lighting, opening fridge, turning on the water heater, opening windows and doors, watering the plants, notifications to feed pets), health care (medicine time alarms, elderly fall detection), and security and surveillance (attacker recognition). In a smart city, vision-based activity recognition can also help to reduce the crime rate by detecting violent actions and sending alarms to police. Additionally, notifying fatigued drivers by detecting their actions can play a role in reducing car accidents. Furthermore, the detection of non-suicidal self-injury based on the spatiotemporal features of indoor activities was another application for human activity recognition [2].

The assessment of ADL is commonly performed in hospitals using standardized methods such as SCIM measure [3]. This procedure requires the caretaker to observe the patient for a long duration to assess if a person can perform the activities. The ADL performance is graded depending on the ability of the person to perform ADL independently, require partial assistance, or require total continuous assistance. Such an assessment takes a long time and is best performed at home so that the human behavior can be naturally observed. In this paper, an effective algorithm to classify videos into a set of pre-defined daily living activities is proposed. The system is capable of automatic video clip classification based on ADL events and thus supports various video analytics for healthcare monitoring. This contributes to more efficient patient monitoring and better diagnosis of a patient’s health status, thus facilitating timely intervention and rehabilitation.

Egocentric vision study images acquired from a first-person or camera-wearer perspective compares the conventional third-person perspective where the image is captured from an external observer. With the proliferation of small portable and wearable cameras, it is now easy to collect a large number of egocentric videos. The video can be used for the semantic understanding of the activities in a naturalistic setting. Egocentric video is useful for many applications related to daily living analysis. It is easy to set up and involves just wearing the camera. However, there are several inherent difficulties and challenges, including continuously moving cameras that lead to motion blur problems. Algorithms that rely on motion patterns are affected by unknown motion dynamics. In a realistic scenario, background clutter and large variations in how the activity is executed pose large intra-class variation. Additionally, an activity performed by numerous persons and in various locations exhibits high variations. Furthermore, different activity classes also share similar appearance patterns, and this leads to small inter-class variance. To address this issue, robust features are needed to discriminate between different activity classes, and these features should be invariant to intra-class variations.

Feature representation plays an important role in many classification tasks. Before a data sample can be classified, each sample must be encoded with suitable features in a fixed dimensional vector. This provides compact numerical representation of the data sample for discriminating between different activity classes. Moreover, a good feature vector allows a simpler classifier to predict accuracies with high generalization, whereas a weak feature vector requires a more complex classifier. This paper studies visual feature representations that are obtained from convolutional neural networks (CNNs). Such a network usually performs well when it is trained with a large image dataset such as ImageNet [4]. To overcome the lack of training samples, many methods use pre-trained CNN for image and video classification. However, such studies in the egocentric video domain are still limited. Egocentric video has unique properties due to the importance of objects that help to predict activity being undertaken. Due to the difference in viewpoint, images in egocentric perspective appear different from the images in the ImageNet dataset. This paper aims to study the utility of features from pre-trained CNN for representing egocentric video frames. A novel CNN-based feature extraction technique that incorporates spatial and temporal pooling is proposed and evaluated. Features are extracted from individual video frames by taking the activation of the last convolution layer. The extracted deep convolutional feature vectors are aggregated with the use of average and max spatial pooling to obtain the proposed MMSP feature. Finally, the MMSP frame feature is integrated with both max and average temporal pooling as well. Experiment results highlight the strong performance of the proposed solution.

Even though there are recent CNNs, VGG network is still a popular deep learner and widely used in many applications. It has shown superior performance at image classification, and it is able to generalize well to the related tasks. VGG-16 is selected because it can balance good performance and fewer parameters.

The contributions of this work are summarized as follows:

1. We evaluated and compared two different sets of features that can be obtained from a pre-trained CNN, namely VGG16. The first feature set includes features from the fully connected layer (fc7), and the second feature set includes features from the last convolutional layer after pooling (fv-pool5).
2. We performed an ablation study to show the advantage of aggregating appearance and motion features for daily activity recognition.
3. We proposed a fusion of two methods, including mean and max spatial pooling (MMSP) and max mean temporal pyramid (TPMM) pooling to compose the final video descriptor.

## 2. Related Works

The use of an egocentric perspective for video-based activity recognition has become increasingly popular due to the widespread use of the wearable camera. The advancement of camera technology resulted in smaller compact devices and longer battery lifespan. This helps to facilitate the use of wearable cameras for activity recognition.

The typical activity recognition pipeline consists of a feature extraction phase followed by classification phase. For example, Internet of Healthcare Things systems have extensively emerged since the Industry 4.0 revolution [5]. They used accelerometers and gyroscope sensors in smartphones to collect the data of handcrafted features obtained from the motion and acceleration signal. Random Forest is used as the classifier to recognize actions. This differs from vision-based activity recognition, where feature extraction is performed on the video frames and is mainly characterized by the types of features used. The important cues for egocentric video classification are the hand pose, objects used, visual features, motion patterns, and gaze points of the scene. This differs from the conventional activity classification from the third-person perspective. In this perspective, activity is observed by an external observer and uses RGB with depth images [6] and skeleton information [7,8].

The activity recognition approaches can be categorized into techniques that use manual feature engineering (handcrafted features) and the more recent methods that use features learned from training data. To extract visual features, the manual feature extraction approach typically uses image-based heuristics that have been known to perform well in image recognition task [9,10]. The same feature extraction technique is applied to extract visual features from the video frames. Motion-based features are borrowed from the one used in action recognition from a third-person perspective (TPR) [11,12]. However, the major problem with the manual feature engineering approach is the need to customize the feature design for different tasks, and this involves a large amount of manual trials and human expertise. The feature-learning approach solves this problem by automatically learning from the training data related to the vision task. This results in a feature vector that is highly discriminative.

Early work on egocentric activity recognition uses handcrafted features adopted from the technique used in the image recognition domain. One of the important works in Daily Activity Recognition was conducted by Pirsiavash and Ramanan [1]. They proposed a challenging dataset (ADL) that shows unscripted daily activities being performed by multiple persons in various locations. To capture the notion that daily living activities are object-based, the presence and absence of key objects are used as discriminative cues. The visual features extracted from each video frame are based on object detection scores for a few relevant objects. The objects are divided into active and passive categories: passive objects appear in the video frame as contextual information while active objects are objects that are manipulated when the activity is being performed by the camera wearer. The use of separate active and passive object detectors is motivated by the distinctive change in appearance when the object is manipulated, and thus change from a passive to active state and then back to a passive state. The object detection scores are aggregated in a two-level temporal pyramid to exploit the temporal ordering of the features. This pioneer work was further improved by [13], which proposed to learn via a boosting method the spatiotemporal partitions of the pyramid that helped to discriminate the activities. This compares to the earlier work by Pirsiavash and Ramanan [1] that assumed equally spaced partitions for the temporal pyramid and did not use spatial partition. However, only a small improvement was observed. This is due to the use of object detectors based on hand-crafted features, which perform poorly on the unconstrained dataset. Another researcher [14] tried to use the salient region predicted from the video frame in the ADL dataset. This salient region attracted gaze points or human attention and thus might contain useful objects that could help in discriminating the activities. Active and passive objects were detected and further classified as salient or non-salient depending on the computed saliency score of the detected regions. This led to a slight improvement in activity recognition and accuracy.

In the work proposed by González Díaz et al. [15], daily activity recognition was performed by relying on an image classification score for a selected set of relevant ADL objects. Object-centric representation was obtained based on the local SURF [16] features aggregated using a Bag of Word method. This is different from the work proposed by Pirsiavash and Ramanan [1], which used a parts-based object detection method that required exhaustive scanning of the whole video frame. Improved performance was observed when a predicted scene category was used and when the aggregated feature was weighted by a saliency map obtained from spatial, temporal, and geometric saliency measures.

Features from a deep convolutional neural network (CNN) pre-trained on a large image dataset such as ImageNet have been shown to be useful as discriminative visual features for many image classification problems [17]. In [18], the visual features were obtained from the first fully connected layer of the CNN network. The model was trained to perform object classification on the ILSVRC13 dataset. The recognition performance using the CNN feature outperformed many state-of-the-art handcrafted feature methods in many image classification problems. This shows that features from pre-trained CNN on a large image dataset can be used as a powerful visual feature for different image classification problems.

The CNN features have also been extended to be used for video classification tasks [19,20]. Jiang et al. [21] used the outputs of the seventh fully connected layer of the AlexNet model [22]. Karpathy et al. [23] used a CNN model that fused features obtained from different video frames and predicted action categories in web-based videos. One interesting result shows that single-frame model performance is comparable to multi-frame model. Zha et al. [24] employed image-trained CNN features from the VGG model [25]. The output of the fully connected and class softmax layer is used as a frame descriptor. Since the number of frames in a video varies, uniform frame sampling was used. The video descriptor was obtained by performing spatial and temporal pooling of the frame-based features.

The CNN features are also used for egocentric video classification. The processing pipeline is similar to video classification for a third-person perspective. The major design issues lie in the selection of different CNN features and the pooling of the frame to form a video descriptor. To incorporate temporal dependencies within video frames, previous techniques applied time series temporal pooling [26] and temporal pyramid of object detection scores [1] to aggregate features to form a fixed-length representation of input to be applied to the classifier. Efficient temporal pooling methods are crucial to preserve salient temporal information from the video frames. Recent work by [27,28] showed that detecting the region where object manipulation takes place is important. This is known as the interactional image region where the activity is observed. The region frequently shows the hand manipulating selected objects related to the activity. Encoding objects and motion-based features from such a region has been shown to provide strong cues for predicting ongoing activity. In the work by Zhou et al. [27], the foreground region related to the activity was detected by combining hand segmentation, salient region detection from CNN convolutional features, and motion maps from the estimated optical flow field. These three feature maps were stacked as a volumetric input sequence to the bounding box regression network based on VGG-16 in order to predict the interactional region. Another paper [28] directly trained an object detector of the Faster-RCNN-ResNet 101 model. The object annotation from the ADL dataset was used. The detected object was tracked at 15 fps and motion features were extracted from the detected object regions in 2 to 3 seconds of video frames. The tracked motion is encoded as a boundary histogram feature (MBH) [11] and a Histogram of Oriented Flow (HOF) [29]. The local motion features obtained from the video were aggregated via Fisher vector encoding to form a final video descriptor for activity classification. Another study employed fine-tuned YOLO-v4 for activity detection and 3D-CNN for recognition using a UCF-Crime dataset [30].

A sensor data contribution analysis method based on status frequency-inverse frequency was proposed for human activity. Spatial distance matrix for context-awareness of cross-activities was found. Novel wide time–domain CNN (WCNN, a powerful parallel feature extraction) was able to save time and produce good performance [31].

Although multiple methods that use CNN for video classification have been proposed, there is still a lack of study on the use of convolutional features for content understanding in egocentric videos. Most existing works used the fully connected layer to obtain video frame features. This representation lacks spatial composition of the visual pattern. On the contrary, features from the convolutional layer give filter activations at relative spatial locations of the image. Additionally, these features are less biased to the concept used in ImageNet dataset. Thus, they can be a useful generic feature for frame-based representation.

In this paper, the use of a pre-trained image CNN for extracting features from egocentric video frame is studied. A new spatial pooling of the convolutional features extracted from a pre-trained CNN is proposed and evaluated. Additionally, a temporal pyramid method is proposed to aggregate the CNN features extracted from the sequence of adjacent video frames. This differs from the work in [1] that requires a number of object detections to be performed for each video frame. Using a bank of object detectors is time-consuming and is sensitive to detection accuracy. Instead of running multiple object detectors, the proposed method uses a pre-trained CNN to extract visual features from each frame, and the features are pooled to form a fixed-length feature vector. The feature vector is used by SVM for multi-class classification in a one-vs.-all configuration.

The organization of this paper is as follows: The introduction to the problem and the data used is described in Section 3. Furthermore, the approaches of CNN-based feature extraction, optical flow, and spatial and temporal pooling are also demonstrated in this section. The description of the three public datasets is described in Section 4. Moreover, Section 4 discusses the experiments conducted in this study and analyzes the results by comparing the proposed method with state-of-the-art methods. Finally, in Section 5, the outcome of this work is summarized to give the readers a glimpse into potential improvements in the future.

## 3. Materials and Methods

In this section, we introduce the problem of activity recognition under study in this work. Furthermore, the proposed method is discussed in detail.

### 3.1. Activity Recognition Problem

Egocentric vision studies images acquired from a first-person or camera-wearer perspective. The camera video can be used for a semantic understanding of the activities in a naturalistic setting. However, there are several inherent difficulties and challenges, including continuously moving cameras that lead to motion blur problems. Algorithms that rely on motion patterns are affected by unknown motion dynamics. In a realistic scenario, background clutter and large variations in how the activity is executed pose large intra-class variations. Additionally, an activity performed by numerous persons and in various locations exhibits high variations. Furthermore, different activity classes also share similar appearance patterns, and this leads to small inter-class variance. To address this issue, robust features are needed to discriminate between different activity classes, and these features should be invariant to intra-class variations. The proposed video features are evaluated on several public datasets. Comparison with selected baseline methods was performed in the video classification framework. Video classification with the proposed features is evaluated on three publicly available benchmark datasets. These datasets are chosen to evaluate the video features in a variety of scenes and activities performed by different people. All the experiments were performed by leave one person out evaluation protocol. The three datasets that have been used are briefly described in Section 4.1.

### 3.2. The Proposed Method

In this section, we will introduce our proposed framework for feature extraction from egocentric video frames. First, we feed video frames into a pre-trained CNN model and obtain the activations from the last convolutional layer. The feature from the video frame is then post-processed with two types of local pooling operations, namely, average pooling and max pooling. Local pooling is used to combine contributions from multiple deep descriptors taken from the output of the convolution layer. The pooling operation is applied to the feature maps at preselected spatial regions. The pooled feature vectors from average and max pooling are concatenated to form the frame descriptor known as MMSP feature. In order to incorporate motion information, optical flow field is extracted from sampled video frames and is then converted to a motion image. The motion image represents the direction and intensity of the optical flow motion in a visual form. The MMSP feature is also extracted from the motion image and evaluated.

To classify the egocentric video clip into corresponding activity classes, the MMSP feature from the sampled frames is extracted and aggregated. The frame features are integrated into a single video descriptor with the use of both max and average pooling, applied in a temporal pyramid scheme. The temporal pooling approach captures motion information on a global and local scale. The final video clip descriptor is known as the MMSP–TPMM feature.

#### 3.2.1. Proposed Video Frame Feature (MMSP)

This section describes how the video frame feature (MMSP) is obtained. It is known that the pre-trained image CNN activations from the last fully connected layer are an effective visual descriptor [18] However, the activation from the fully connected layers does not provide information on the spatial arrangement of the image parts. Such information is essential for discriminating between different types of scenes and objects related to ADL. Thus, features from the last convolutional layer are proposed. The features are obtained from the activations of the pre-trained CNN model, namely, VGG16. The VGG16 model has been pre-trained on the large ImageNet dataset used for the ILRSVRC 2014 competition for predicting 1000 image categories.

**Figure 1:**
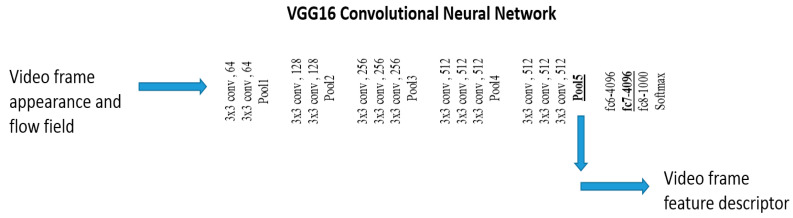
Block diagram of the VGG16 CNN model used to extract features from video frame and flow image separately by using two VGG16 networks.

**Figure 2:**
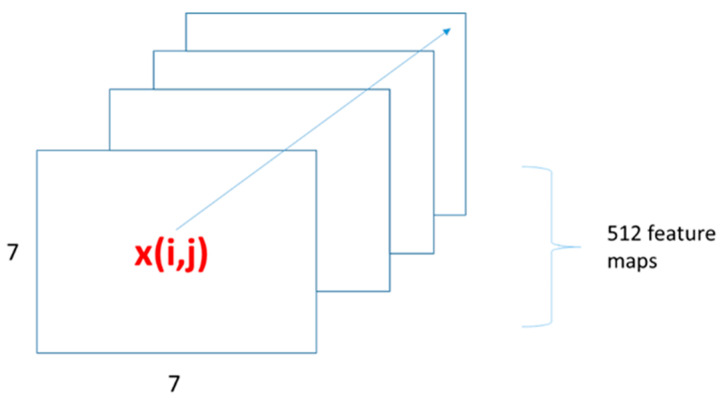
Diagram of the output from the Pool5 convolutional layer. The output is a stack of 512 feature maps. The feature maps consist of 49 (7 × 7) convolutional features, and each feature $x\left(i,j\right)$ at location $\left(i,j\right)$ is a 512-dimensional vector.

The VGG16 CNN image model has 16 layers. It contains thirteen convolutional layers and three fully connected layers. The convolutional layers are arranged into five blocks. Each block consists of a sequence of 3 × 3 convolutional operations on the input feature maps. The block diagram in Figure 1 shows the sequence of convolutional layers, interleaved with pooling layers after each block. The convolutional features were obtained from the Pool5 layer. For each input image, the Pool5 layer emits a stack of 512 feature maps with 7 × 7 spatial dimensions. The feature maps are illustrated in Figure 2. The feature maps represent the 512 filter activations at various spatial locations of the image. The activations are organized as 7 × 7 cells, and each cell at location $\left(i,j\right),\;i,j\;\in \;\left[1,7\right]$ consists of a 512-dimensional deep convolutional feature $x\left(i,j\right)\;\in \;R^{512}$.

**Figure 3:**
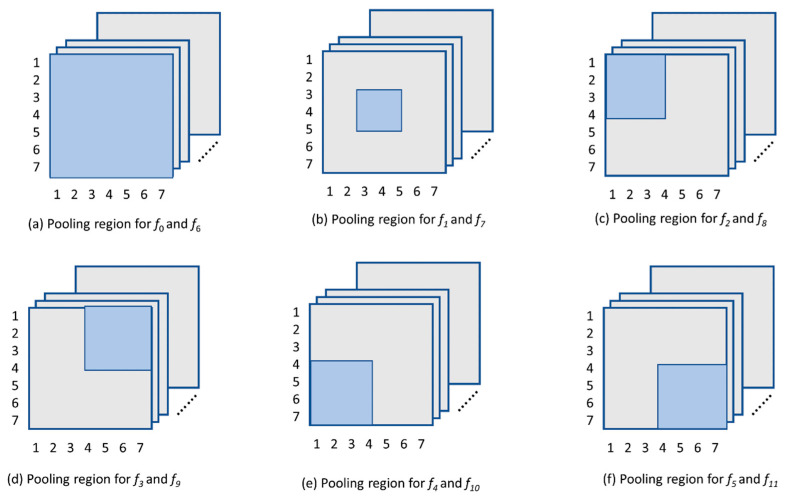
Definition of the areas (coloured region) used as local pooling regions in Equations (1)–(12). These regions are used for both mean and max pooling operations. The pooling operation is applied to every feature map.

Stacking the convolutional feature to form a single vector as a video frame descriptor will produce a vector with a high dimension of 25,088 (512 × 49). In the proposed technique, the large dimensional vector space is reduced by using spatial pooling at multiple fixed regions of the feature maps. The local pooling regions are shown in Figure 3. The pooling regions are selected to cover the five major spatial areas, namely the center, top left, top right, bottom left, and bottom right areas. The series of Equations (1)–(15) shown in Table 1 explain the heuristic method used to extract visual features from the input video frames. The mean and max pooling operations are applied to the specified regions in Figure 3 with the use of Equations (1)–(12). Equations (13) and (14) are then applied to concatenate the resulting feature vectors. The final video frame descriptor known as MMSP is obtained by concatenating the result of both mean and max pooling operations, as shown in Equation (15). Pooling is performed element-wise for each channel and thus produces one convolutional feature vector with 512 dimensions for each pooling region. This effectively captures the representative features for each filter channel and reduces the number of convolutional feature vector from 49 to 12. The total dimension of the MMSP video frame feature vector is 6144 (12 × 512).

The description of each equation in Table 1 used to extract visual features from the input video frames utilizing heuristic method is as follows:

Equation (1) performs spatial average pooling by averaging all 49 convolutional feature vectors in an element-wise manner to produce a single 512-dimensional feature vector. The region covers the whole image and is indicated in Figure 3a.

Equation (2) performs spatial average pooling by averaging the middle spatial region convolutional feature vectors, as indicated in Figure 3b.

Equation (3) performs spatial average pooling by averaging the top left spatial region convolutional feature vectors, as indicated in Figure 3c.

Equation (4) performs spatial average pooling by averaging the top right spatial region convolutional feature vectors, as indicated in Figure 3d.

Equation (5) performs spatial average pooling by averaging the bottom left spatial region convolutional feature vectors, as indicated in Figure 3e.

Equation (6) performs spatial average pooling by averaging the bottom right spatial region convolutional feature vectors, as indicated in Figure 3f.

Equations (7)–(12) similarly applied spatial max pooling in the mentioned regions described in Figure 3. Equations (13)–(15) combine the result from previous equations in order to obtain the final MMSP video frame descriptor.

In this study, the activity motion pattern is represented by dense optical flow pattern. Optical flow is the perceived motion of objects in a visual scene. In order to obtain the flow image, the dense optical method [32] is converted to an image representation in order to obtain the flow image [33]. The image hue value represents the direction of motion, and the saturation intensity value represents the magnitude of motion.

**Figure 4:**
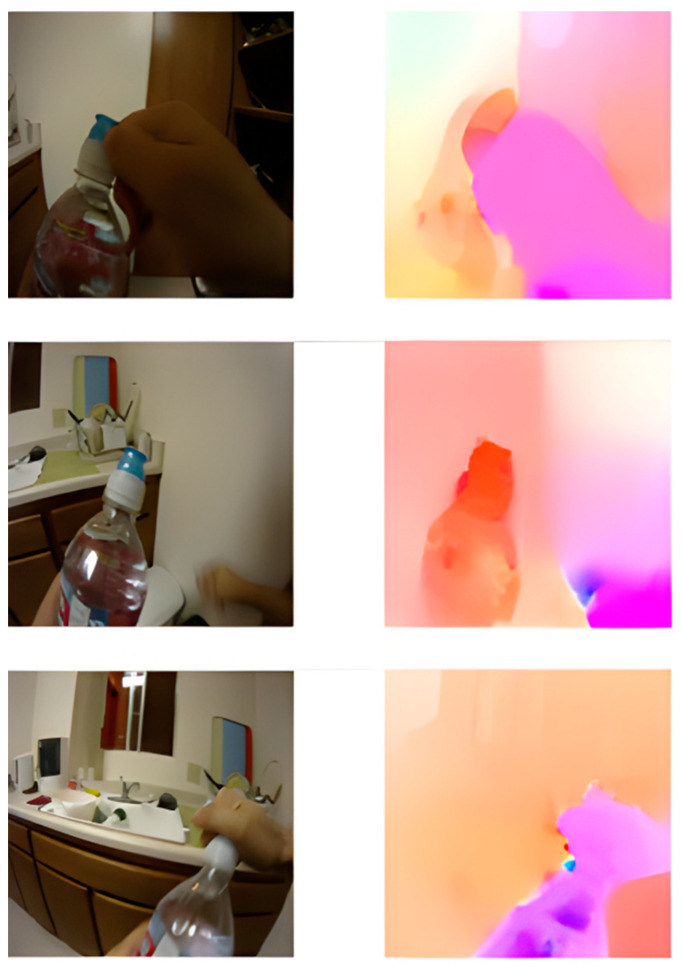
Visualization of frame image and motion image for ‘drinking water’. The motion image is obtained from optical flow field converted to image.

The VGG16 CNN model is used to extract the visual features from the motion image. Figure 4 shows sample visual frames and their corresponding flow image obtained from dense optical flow estimation. It can be observed that strong motion detected in the frame corresponds to the foreground region. The MMSP frame feature is applied to the flow image as well. The fusion of features from frame image and flow image gives a total dimension of 12,288 dimensions (6144 × 2).

Previous research by [34] has shown that aggregating the CNN convolutional features by average and max pooling leads to the best accuracy in fine-grained image classification when compared to other feature aggregation techniques. Average pooling combines the contribution from all features in the selected spatial area, and max pooling only chooses the feature with the strongest activation. Strong activation indicates the presence of objects in the represented locations. Average pooling considers objects placed at different locations in the scene, and max pooling considers the most prominent object in the scene. Our proposed method uses multiple fixed local regions, whereas Wei et al. [34] only used one most salient region for the max and average pooling. By performing regional spatial pooling, object representation located at different positions can be captured.

#### 3.2.2. Proposed Video Clip Feature (MMSP–TPMM)

Videos contain a different number of frames in a sequence of different lengths. To encode the video frames, temporal pooling of the individual MMSP frame features is used. Recent work on video classification showed that not all frames are needed, and only a small number of relevant frames in 1.5 s period are sufficient [35]. The CNN features from individual frames can be aggregated by a few standard approaches, namely, average pooling, max pooling, and temporal pyramid pooling. The mean pooling method aggregates the contribution of all frames by taking the average value from all video frames. The max pooling method chooses the feature from the frame with maximum signal strength. The definition of the proposed temporal pyramid max–min (TPMM) pooling is described as follows:

Let $T^{j,k}$ denote the temporal segment number $j,\;j\in \{1,\ldots ,2^{k-1}\}$ in the temporal pyramid level $\;k,\;k\in \left\{1,2\right\}$ for a two-level pyramid. Consider a set of consecutive frames in the video clip $V_{i}=\left\{f_{1},\;f_{2}\;,\;\ldots ,\;f_{N_{i}}\right\}$, where $N_{i}$ represents the number of selected frames in the video clip $V_{i}$ and $f_{t}$ denotes the ${\;f}_{}^{mmsp}$ feature for the video frame *t*, $t\in \left[1,N_{i\;}\right]$.

**Figure 5:**
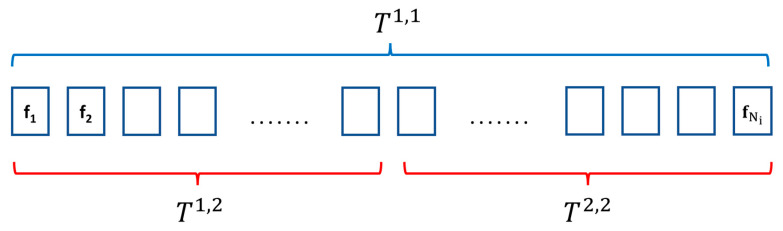
The diagram shows the division of frames in a video clip *i* with length N_i_. In the first level pyramid *T*^1,1^, max pooling is applied over all the frames in the video clip, based on Equation (16). In the second level pyramid, the video frames are divided equally into two temporal segments *T*^1,2^ and *T*^2,2^. The mean pooling operation is applied to the first half and second half of the video frames in the given segment (Equations (17) and (18)).

**Figure 6:**
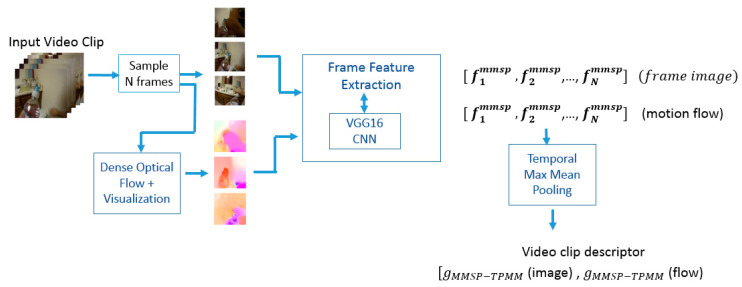
Process flow in extracting video clip feature vector. The same pre-trained CNN model VGG16 was utilized for the extraction of the discriminative visual patterns from the sampled video frame and flow image. The temporal pooling operator was applied separately for the video frame and flow image channel.

To convert a sequence of frame features into a single feature vector as video descriptor, temporal pyramid pooling is proposed. Figure 5 illustrates the temporal pyramid pooling scheme used. The temporal pooling operation is defined in Equations (16)–(19), and the concatenate is the result of mean and max pooling to form the video clip descriptor. Two levels of temporal scale are used. In the first level ${\;T}^{1,1}$, temporal max pooling is performed over the whole video clip. In the second level, the video clip is divided into two equal segments, and temporal mean pooling is applied to each segment. The frame division into corresponding temporal segments in the scheme is detailed as $T^{1,1}=\left[1,N_{i}\right]\;,\;T^{1,2}=\left[1,\frac{N_{i}}{2}\right],\;\mathrm{and}\;T^{2,2}=\left[\frac{N_{i}}{2}+1,N_{i}\right]$. The video clip descriptor is defined as $g_{MMSP-TPMM}$ in Equation (19) and named MMSP–TPMM video feature. The L2 normalization is applied to the video features before being applied to the SVM multi-class classifier. The process flow for the video clip feature extraction is shown in Figure 6.

(16) $$g_{max}^{0}\left(V_{i}\right)=\underset{t\in [1,N_{i}]}{\max}f_{t}$$

(17) $$g_{mean}^{1}\left(V_{i}\right)=\frac{1}{{(N}_{i}/2)}\sum_{t\in [1,N_{i}/2]}f_{t}$$

(18) $$g_{mean}^{2}\left(V_{i}\right)=\frac{1}{{(N}_{i}/2)}\sum_{t\in [\frac{N_{i}}{2}+1,\;N_{i}]}f_{t}$$

(19) $$g_{MMSP-TPMM}=[g_{max}^{0}\left(V_{i}\right)\;,\;g_{mean}^{1}\left(V_{i}\right),\;g_{mean}^{2}\left(V_{i}\right)]$$

Indeed, we used VGG16 without fully connected layers, and as a result, it has few parameters. We replaced fully connected layers using a linear support vector machine (SVM). The feature vector is applied to SVM classifier for multi-class classification. The reason for this replacement is not only to increase speed but also to enhance performance because the features of fully connected layer (fc7) are more biased towards the prediction of the 1000 categories of ImageNet dataset.

The dimension of the video feature produced by Equation (19) is fixed for every video dataset and includes 18,432 (6144 × 3) for a single modality. In the case where features from both spatial images and flow images are used, the dimension for the video feature will be doubled (18,432 × 2).

## 4. Experiments and Results

In this section, a description of the three public datasets is highlighted. Additionally, we discuss the experimental setup of the conducted experiments. Furthermore, the performance metrics used for evaluation are described. Finally, the experimental results of the proposed method are demonstrated to show the superior performance of the proposed solution.

### 4.1. Datasets Overview

The proposed video features are evaluated on several public datasets. A comparison with selected baseline methods was performed in a video classification framework. Video classification with the proposed features is evaluated on three publicly available benchmark datasets. These datasets are chosen to evaluate the video features in a variety of scenes and activities performed by different people. All the experiments were performed with the use of leave one person evaluation protocol. The three datasets that have been used are briefly described as follows:

1. Activity of Daily Living (**ADL**) dataset [1]: The dataset contains activities performed by 20 people in their own homes. A camera was worn on the chest, and videos were captured at 30 frames per second with a 170-degree viewing angle and with a high-definition resolution setting (1280 × 960). Each person recorded the video for roughly 30 min. Thus, the total frames per video clip are 30 × 60 × 30 = 54,000. Activities were performed freely without being scripted. The unconstrained video capture condition resulted in a wide diversity in the captured video in both the scenes and objects used in the activities. This makes the video clip classification more challenging when compared with other egocentric video dataset. For benchmarking with the experiment setup in [1], 18 activities by 13 people (persons 7–20) from the ADL dataset were selected for experimental evaluation. The summary of the activities used in the video classification experiment is shown in Table 2. It can be observed that the distribution of the video classes is imbalanced. The activity duration and number of video clips for the class differ significantly.

2. Life-logging egocentric activities (**LENA**) dataset [36]: The dataset was prepared with activities performed by 10 people. Each person was asked to perform 13 different activities, and each activity was repeated twice. This yields a total of 260 video clips, and each of the videos was annotated with a single activity label. The duration of the video clips in this dataset is 30 s, and they were recorded at 30 frames per second with downscaled resolution of 430 × 240. Each video clip has 30 × 30 (900) frames. The videos were recorded with the use of a special augmented spectacle known as Google Glass. The dataset is organized into two levels. The top level shows the general activity types, and each contains a set of more specific activities. The distribution of the activities in this dataset is shown in Table 3.

3. First-Person Personalized Activities (**FPPA**) dataset [37]: This dataset contains 586 videos recorded by five subjects with a Go-Pro camera mounted on the head. Two of the subjects live in separate homes, and three subjects share the same home. The scene is less diverse when compared to the ADL dataset since three of the persons share the same home. Each video lasted for approximately 10 s and was recorded at 30 frames per second with a resolution of 1280 × 720. Each video clip has 10 × 30 (300) frames. A total of five activities were prepared, namely, drinking water, putting on shoes, using the refrigerator, washing hands, and putting on clothes. As shown in Table 4, the activities in the dataset have a balanced distribution.

### 4.2. Experiment Setup

The performance of the proposed solutions was evaluated by conducting four different experimental scenarios. The ADL dataset is the main benchmark for performance validation. All experiment codes were implemented using MATLAB software and the deep-learning tool from MatConvNet [38]. The description of each experiment is listed in the following section.

Four different experiments were implemented to evaluate the proposed MMSP–TPMM features. The effectiveness of the video feature configurations for capturing spatial, temporal, and motion patterns was assessed. The proposed features were used for egocentric video classification tasks and utilized the datasets described in Section 3.1. Experiments were conducted to measure the performance of the proposed MMSP–TPMM video descriptor against alternative feature settings and a temporal pooling strategy. In addition, the benefit of fusion with both appearance and motion flow images was evaluated. The linear support vector machine (SVM) with a one-vs.-all multi-classification method was used as the classifier. The Liblinear software from [39] was used with the SVM misclassification cost parameter C set to 1. It was found through multiple trials that SVM was not sensitive to the value of C, and the value C = 1 gave consistent satisfactory results.

### 4.3. Performance Metrics

Activity class prediction performance was measured based on multi-class classification accuracy and average per-class precision (AP). The evaluation was performed with leave one person out (LOPO) cross-validation scheme. The video clips left out were used for testing, and all the remaining video samples were used for training. This was repeated to ensure each person in the dataset was left out for validation of the trained classifier. The confusion matrix entry $C_{ij}$ was populated for each classified test sample. The row of the matrix refers to the ground truth label while the column is the predicted class. Each entry $C_{ij}$ stores the number of samples with the predicted class j, but the actual class is i. Let N be the number of validated samples and $N_{c}$ the total number of classes used. The overall accuracy is computed by Equation (20), which shows the ratio of correct class predictions over the number of validation samples used. The multi-class classification scheme can be formulated as a binary classification for each class in the validation set. This is achieved via a one-vs.-all classification scheme. The precision shows the ratio of correct positive class predictions for the target class over the total positive predictions made, as shown in Equation (21). Thus, the precision for each class *j*, Pre(j), can be calculated as in Equation (22). The average per-class precision AP is computed as seen in Equation (23):

(20) $$\;Accuracy\;(\%)=100\times \sum_{i=1}^{N_{c}}C_{ii}/N\;\;$$

(21) $$Precision\;(\%)=100\times \frac{TP}{TP+FP}$$

(22) $$Pre(j)(\%)=100\times \frac{C_{jj}}{\sum_{i=1}^{N_{c}}C_{ij}}\;$$

(23) $$AP\;(\%)=\frac{100}{N_{c}}\;\sum_{j=1}^{N_{c}}Pre(j)\;\;\;\;\;\;$$

### 4.4. Results and Discussion

In this section, the description of each experiment is discussed. Furthermore, the performance metrics of the proposed method and the comparison with state-of-the-art methods are highlighted in detail.

#### 4.4.1. Experiment 1: Video Frame Feature Evaluation

The first experiment evaluates the proposed video frame appearance feature, described as the MMSP feature. Videos from the ADL dataset are used for evaluation, and motion information is not used. The MMSP feature is compared with the feature obtained from the last fully connected layer before the classification layer of the pre-trained VGG-16 model. The feature is simply named fc7. The fully connected layer fc7 has a dimension of 4096 and is applied to L2 normalization before classification. Comparison with the concatenation of the features from the Pool5 convolutional layer (fv-pool5) without the proposed spatial pooling method is performed as well. This yields a feature size of 25,088 (49 × 512). To obtain the final video descriptor, all the video frame features are pooled by temporal max pooling to aggregate features from all frames in the video clip. The VGG16 CNN model was able to extract the features at an approximate time of 0.25 s per frame using the Graphical Processing Unit (GPU) Nvidia RTX3060. The total time to extract the feature from the whole video depends on the number of frames in the video. So, the total time = number of frames × 2 (frame + flow image) × 0.25 (time of feature extraction per frame).

The performance of the video descriptor is evaluated in the video classification task. Classification accuracy and average precision are used as performance metrics. The baseline result for the ADL dataset was reported by [1]. The cited approach used an object detection score as a feature. The fusion between MMSP and fc7 feature is used for comparison. The results of the video classification performance of the compared frame features are shown in Table 5.

The first experiment results on the ADL dataset in Table 5 show a significant improvement of the CNN frame features (fully connected and convolutional) when compared with the baseline method [1] that used object detection scores. The object detector used the deformable part models with SVM [40]. The model was trained with 24 ADL-related object categories, with roughly 1200 training instances per category. The MMSP features gave higher accuracy and average per-class precision when compared to the fc7 feature and by stacking the fv-pool5 features. This shows that MMSP features effectively capture important object representation in the egocentric scene even though the feature dimension is much lower (6144 compared to 25,088).

The CNN model (VGG16) has been trained on the large ImageNet dataset for the ILSVRC 2014 image classification challenge. The types of images are mainly web and photographic images. The fully connected layers provide a feature activation that is more tuned to the images found on the Internet. In this type of image, the object is prominently visible and covers a significant portion of the image. The fully connected layer combines the convolutional feature to discriminate between the 1000 classes of images in the ILSVRC 2014 challenge dataset. Thus, the fc7 feature is more biased towards the prediction of the 1000 class categories. In an egocentric video, the appearance of the object is smaller, and typically, there are multiple objects present in the scene. These object-like features are localized and represented as convolutional features. The convolutional features encoded with the MMSP method have higher performance since they provide a semantically richer representation by fusing the max and mean spatial pooling. Good classification results suggested that the MMSP feature can provide a more discriminative visual pattern representation for ADL activities in the egocentric video.

#### 4.4.2. Experiment 2: Evaluation on Temporal Pooling Methods

In experiment 2, the benefit of the proposed temporal pooling method is evaluated. All frames used the MMSP features, and video sequences are down-sampled to one frame per second. The proposed TPMM pooling method is used for temporal pooling, and the resulting video descriptor is named MMSP–TPMM. The TPMM pooling is compared with the max and mean pooling approaches. In addition, the temporal pooling method by [26] is implemented and tested for comparative assessment. The pooling method is known as Pooled Time Series (PoT), where a sequence of frame features is represented as a multi-variate time series. In the PoT method, a combination of four pooling operators is used, and these include the max, sum, gradient sum, and gradient count. The gradient sum computes the sum of the number of positive and negative increments of the time series, whereas the gradient count computes the total count of positive and negative increments. The details of the formulation of the pooling operators can be found in [26]. Classification performance on the ADL dataset is shown in Table 6.

In the second experiment, the results listed in Table 6 show the benefit of the temporal multi-scale approach used in TPMM pooling. The use of max mean temporal pyramid pooling exceeded the performance of using either max or mean pooling alone over the entire video clip. This shows the complementary effect of both max and mean pooling methods when used in a multi-scale temporal arrangement. A single max temporal pooling operator itself can outperform the more complex Pooled Time Series (PoT) method. This implies a high redundancy among the MMSP frame features in the video clip. Additionally, a max pooling operator can capture the features from the most important frame.

#### 4.4.3. Experiment 3: Evaluation on Multimodal Video Features

The third experiment measured the impact of using both motion flow images and video frame images in the egocentric video classification performance. The same video frame encoding with the MMSP feature and TPMM for temporal pooling of the video frames feature is used (MMSP–TPMM). The MMSP–TPMM video descriptor utilized features from both frame appearance and flow image that are concatenated in an early fusion setting. Frame rates of 1 fps and 10 fps are used to study the effect of frame rate. Classification performance on the ADL dataset is shown in Table 7.

We conducted an ablation study to demonstrate the effect of removing either appearance or flow features using the ADL dataset. The results in Table 7 show that frame appearance features perform better than the flow image features. This is due to the view of objects that strongly correlate with the activity. Both used MMSP features and TPMM temporal pooling. On the other hand, the fusion of flow and appearance images encoded with MMSP–TPMM features as a video descriptor has been found to produce the best video classification results. The result shows that the temporal video patterns are well captured by the proposed method. Interestingly, the performance is consistent for both a low frame rate of 1 fps and a higher frame rate of 10 fps.

Next, we compare our solution with several methods used for egocentric video classification on the ADL dataset. The proposed MMSP–TPMM method with a fusion of both appearance and flow images yielded the best results in terms of the average precision score when compared to several compared methods. The details of the comparison are shown in Table 8. The second best method in terms of performance is by [28]. They employed the Faster-RCNN method to detect a set of objects and encode detected object movement with MBH (motion boundary histogram) features. This is followed by the aggregation of the local features by Fisher encoding to obtain the final video descriptor. Compared to them, we use convolutional features to represent the latent concepts in the video frames. The frame features are then aggregated via temporal pyramid pooling.

#### 4.4.4. Experiment 4: Evaluation on LENA and FPPA Dataset

Additional experiments on the LENA and FPPA datasets were performed to assess if the good results obtained in the ADL dataset can be replicated in different datasets. The proposed method of the MMSP–TPMM feature on both appearance and flow image was also evaluated on the LENA and FPPA datasets. The reported classification accuracies using the handcrafted features of Histogram of Oriented Flow (HOF), Histogram of Oriented Gradient (HOG), Motion Boundary Histogram (MBH), and trajectory features were compared. The results are shown in Table 9. We perform a comparison with the results reported in [36] that used handcrafted features for activity representation. The LOPO cross-validation evaluation method is applied. Frame feature temporal pooling is evaluated on the FPPA dataset.

The experiments’ results in Table 9 and Table 10 confirm the benefit of the proposed MMSP–TPMM features. It outperformed the handcrafted features in the LENA dataset and PoT features in the FPPA dataset. Here, the PoT method was applied to MMSP frame features. Video prediction performance in terms of multi-class classification accuracy and average precision was significantly higher in the LENA and FPPA datasets when compared to the ADL dataset. This is due to the more constrained scene and lower image diversity in these two datasets.

## 5. Conclusions

In this paper, a novel fusion method was proposed to aggregate appearance and motion features and then combine mean and max spatial pooling (MMSP) with max mean temporal pyramid (TPMM) pooling to compose the final video descriptor. We targeted egocentric videos that involve activities of daily living. In such activities, the manipulation of objects is an important cue. The feature representation is designed to capture generic visual patterns that represent the fine-grained object parts and the spatial arrangement of these parts in the image. To extract motion, the technique of converting an optical flow field into an image representation is used. The pre-trained CNN (VGG16) is utilized to extract visual features for both video frames and optical flow images. The spatial and temporal redundancies of visual contents in the egocentric video are exploited by using mean and max spatial pooling (MMSP) for the frame features and max mean temporal pyramid (TPMM) pooling for the video clip. The proposed video descriptor MMSP–TPMM is evaluated in several experiments on three public benchmark datasets. Additionally, comparisons with alternative feature configurations and handcrafted feature methods are made. All the results show improvement in activity classification performance when the proposed solution is used. The fusion of frame appearance and motion features also shows classification performance enhancement, even though the egocentric video is unstable.

Although the proposed method for video features works well when compared to competing methods, there are several limitations. Firstly, the max pooling method will only take the maximum value of the feature over a fixed spatial region. If there are multiple features that are highly activated, only the feature with the maximum value will be taken. This will ignore contributions from other features. For the case of mean pooling, the average value of the feature is influenced by non-important background objects. This will reduce the discrimination strength of the feature. The pooling region is fixed at various spatial regions for the frame feature, and only three different fixed temporal regions are used. A more adaptive temporal pooling scheme may yield a higher discriminative video descriptor.

Given the efficiency of the proposed method, the current limitation is that the model should run on a powerful machine with a large RAM size to consider the model’s parameters and with GPU to obtain high-speed inference. In other words, the current method in its current status cannot run on an embedding system such as a Smartphone with limited RAM and a small processing unit. For future work, we propose replicating the same approach with more computationally efficient CNNs, such as MobileNet or EfficientNetB0, to balance accuracy and inference speed so that we can run the proposed method on an edge-computing IOT for real use of ADL system. Furthermore, we plan to improve the performance of the solution by training a recent pre-trained vision transformer to evaluate their abilities to focus more attention on the targeted objects.

Additionally, employing the proposed method in other real-life applications, such as medical image analysis, can contribute to improving the analysis performance by enhancing the detection of lung cancer tumors in CT scan imaging, considering the motion that needs to be estimated and corrected [40].

## Figures and Tables

**Table 1:** A set of equations used to extract visual features from the input video frames utilizing heuristic method.

$f_{0}=\frac{1}{49}\sum_{i=1}^{7}\sum_{j=1}^{7}x\left(i,j\right)$	(1)
$f_{1}=\frac{1}{9}\sum_{i=3}^{5}\sum_{j=3}^{5}x\left(i,j\right)$	(2)
$f_{2}=\frac{1}{9}\sum_{i=1}^{4}\sum_{j=1}^{4}x\left(i,j\right)$	(3)
$f_{3}=\frac{1}{9}\sum_{i=1}^{4}\sum_{j=4}^{7}x\left(i,j\right)$	(4)
$f_{4}=\frac{1}{9}\sum_{i=4}^{7}\sum_{j=1}^{4}x\left(i,j\right)$	(5)
$f_{5}=\frac{1}{9}\sum_{i=4}^{7}\sum_{j=4}^{7}x\left(i,j\right)$	(6)
$f_{6}=\;\underset{i\in \left[1,7\right],\;j\in [1,7]}{\max}\left\{x(i,j)\right\}$	(7)
$f_{7}=\;\underset{i\in \left[3,5\right],\;j\in [3,5]}{\max}\left\{x(i,j)\right\}$	(8)
$f_{8}=\;\underset{i\in \left[1,4\right],\;j\in [1,4]}{\max}\left\{x(i,j)\right\}$	(9)
$f_{9}=\;\underset{i\in \left[1,4\right],\;j\in [4,7]}{\max}\left\{x(i,j)\right\}$	(10)
$f_{10}=\;\underset{i\in \left[4,7\right],\;j\in [1,4]}{\max}\left\{x(i,j)\right\}$	(11)
$f_{11}=\;\underset{i\in \left[4,7\right],\;j\in [4,7]}{\max}\left\{x(i,j)\right\}$	(12)
$\begin{array}{ccc} & f_{mean}= & {\left[f_{0},f_{1},\ldots f_{5}\;\right]}^{T} \end{array}$	(13)
$\begin{array}{ccc} & f_{max}= & {\left[f_{6},f_{7},\ldots f_{11}\;\right]}^{T}\;\;\;\;\;\;\;\;\;\;\;\;\;\;\;\;\;\;\;\;\;\;\;\;\; \end{array}$	(14)
$\begin{array}{ccc} {f_{}^{mmsp}=\left[\;f_{mean,}f_{max}\;\right]}^{} & & \end{array}$	(15)

**Table 2:** Summary of Activity of Daily Living (ADL) dataset statistic used for video classification evaluation.

No.	Activity Type	Number of Clips	Average Number of Frames Per Clip	Average Video Clip Duration (s)
1	‘combing hair’	7	896	29.9
2	‘make up’	3	1350	45.0
3	‘brushing teeth’	10	2613	87.1
4	‘dental floss’	2	1065	35.5
5	‘wash hands/face’	25	734	24.5
6	‘dry hands/face’	31	234	7.8
7	‘laundry’	10	5304	176.8
8	‘washing dishes’	23	2216	73.9
9	‘making tea’	14	1948	64.9
10	‘making coffee’	4	4748	158.3
11	‘drinking water/bottle’	13	1260	42.0
12	‘drinking water/tap’	2	435	14.5
13	‘making cold food/snack’	6	1925	64.2
14	‘vacuuming’	5	3204	106.8
15	‘watching tv’	16	5312	177.1
16	‘using computer’	16	3932	131.1
17	‘using cell’	10	1209	40.3
18	‘reading book’	6	4690	156.3

**Table 3:** Summary of videos in LENA dataset.

Top Level	Second Level	Number of Videos
Motion	Walk straight	20
	Walk back and forth	20
	Walk up and down	20
	Running	20
Social interaction	Talk on the phone	20
	Talk to people	20
Office work	Watch videos	20
	Use Internet	20
	Write	20
	Read	20
Food	Eat	20
	Drink	20
Housework	Housework	20

**Table 4:** Video categories in FPPA dataset.

No.	Categories	Number of Samples
1	Drinking water	116
2	Putting on clothes	108
3	Putting on shoes	113
4	Using the refrigerator	132
5	Washing hands	117

**Table 5:** Multi-class classification accuracy for egocentric activity recognition on ADL dataset. The pretrained deep CNN model VGG16 is used for feature extraction.

Method	Accuracy (%)	AP (%)
Bag-of-objects + Active object model [1]	36.95	36.31
Fully connected layer fc7 (Dimension = 4096)	59.11	42.79
Stacking fv-pool5 convolutional feature (Dimension = 25,088)	62.07	47.40
Proposed MMSP feature (Dimension = 6144)	66.50	51.05
**MMSP feature + fc7**	**67.00**	**51.40**

**Table 6:** Evaluation of proposed temporal pooling method on ADL dataset. The MMSP feature is used as frame feature.

Method	Accuracy (%)	AP (%)
Pooled Time Series (PoT) [26]	64.04	47.74
Mean temporal pooling	59.11	44.11
Max temporal pooling	66.50	51.05
**MMSP–TPMM feature (ours)**	**68.97**	**51.92**

**Table 7:** Evaluation of ADL dataset with appearance image and optic flow image.

	1 fps Frame Rate		10 fps Frame Rate	
Method	Accuracy (%)	AP (%)	Accuracy (%)	AP (%)
MMSP–TPMM on motion flow image (feature B)	59.11	43.04	58.62	43.78
MMSP–TPMM on video frame (feature A)	68.97	51.92	69.95	53.13
Early fusion of features A and B by feature concatenation	**75.37**	**58.42**	**73.4**	**58.19**

**Table 8:** Comparative evaluation of ADL dataset for activity classification.

Methods	AP (%)
Bag-of-Micro-Actions with MBH [28]	57.14
CNN Object and motion feature [27]	55.20
Boost-RSTP + OCC [13]	38.70
Bag-of-objects + Active object model [1]	36.31
**MMSP–TPMM pooling (ours)**	**58.42**

**Table 9:** Average multi-class classification accuracy of LENA dataset with MMSP–TPMM features for video classification results was compared with handcrafted features reported in [36].

Methods	Average Accuracy
HOF [36]	84.00%
HOG [36]	77.42%
MBH [36]	82.46%
Trajectory [36]	84.50%
Combined HOG, HOF, MBH, and Trajectory [36]	84.23%
**MMSP–TPMM pooling (Ours)**	**90.38%**

**Table 10:** Average multi-class classification accuracy on FPPA dataset.

Methods	Average Multi-class Accuracy	Average Per-Class Precision
MMSP–TPMM (Appearance only)	92.83%	92.93%
Point of time series (PoT) [26]	94.54%	94.59%
**MMSP–TPMM** **(Appearance and motion, ours)**	**96.08%**	**96.11%**

## Data Availability

All datasets used are from the public domain and available from the relevant author’s website as indicated in the following link: [ADL dataset https://redirect.cs.umbc.edu/~hpirsiav/papers/ADLdataset/ (accessed on 1 December 2019), FPPA dataset http://tamaraberg.com/prediction/Prediction.html (accessed on 1 December 2019), LENA Dataset http://people.sutd.edu.sg/~1000892/dataset (accessed on 1 December 2019) ].
